# LACP-SG: Lightweight Authentication Protocol for Smart Grids

**DOI:** 10.3390/s23042309

**Published:** 2023-02-19

**Authors:** Muhammad Tanveer, Hisham Alasmary

**Affiliations:** 1 Department of Computer Science, University of Management and Technology, Lahore 54770, Pakistan; 2 Department of Computer Science, College of Computer Science, King Khalid University, Abha 61421, Saudi Arabia

**Keywords:** authentication, smart grid, AEAD, privacy, session key, ROM

## Abstract

Smart grid (SG) recently acquired considerable attention due to their utilization in sustaining demand response management in power systems. Smart meters (SMs) deployed in SG systems collect and transmit data to the server. Since all communications between SM and the server occur through a public communication channel, the transmitted data are exposed to adversary attacks. Therefore, security and privacy are essential requirements in the SG system for ensuring reliable communication. Additionally, an AuthentiCation (AC) protocol designed for secure communication should be lightweight so it can be applied in a resource-constrained environment. In this article, we devise a lightweight AC protocol for SG named LACP-SG. LACP-SG employs the hash function, “Esch256”, and “authenticated encryption” to accomplish the AC phase. The proposed LACP-SG assures secure data exchange between SM and server by validating the authenticity of SM. For encrypted communication, LACP-SG enables SM and the server to establish a session key (SEK). We use the random oracle model to substantiate the security of the established SEK. Moreover, we ascertain that LACP-SG is guarded against different security vulnerabilities through Scyther-based security validation and informal security analysis. Furthermore, comparing LACP-SG with other related AC protocols demonstrates that LACP-SG is less resource-intensive while rendering better security characteristics.

## 1. Introduction

The Industrial Internet of Things (IIoTs) promises to elevate many communication paradigm innovations, focusing on industrial applications. Particularly, IIoT-based smart grid (SG) technology is envisioned to be a vital part of the next-generation power grid system. An SG mainly comprises four elements: sensing, control, actuation, and communication systems. The sensing and communication processes are performed by smart meters (SMs), which are the significant components of an SG, while service providers perform actuation and communication (SPs) [1].

The rapid utilization of SMs has recently been witnessed in smart homes under the SG environment to observe energy utilization in real time. To this end, the SMs communicate with SP on public communication channels. The communication between SMs and SP mandates security and privacy, as the channel used for this communication is prone to various security risks. For instance, an adversary can modify, eavesdrop, and disrupt the communication with consequent degradation in the performance of the SG system [2]. These concerns necessitate the designing of a secure, lightweight, and robust authentication (AC) protocol to guarantee information communication among the honest participants in the SG system while preserving the privacy of the entities.

### 1.1. Security Requirements in SG Systems

An SM transmits electricity usage information periodically to SP via the public internet. Therefore, the following security requirements are imperative for the smooth working of the SG system [3,4].

#### 1.1.1. Security

Firstly, the SG system contains a large number of SMs. Thus, an SP must check the authenticity of the SM before commencing the information exchange process. It is worth noticing that, by authentication, the authenticity of the deployed SMs in the SG system can be verified. Therefore, the authentication protocol should be able to resist various security attacks, such as denial-of-service (DoS), SM capture, ephemeral secret leakage (EPSL), device impersonation (DIMP), man-in-the-middle (MIDM), de-synchronization (DeS), privilege-insider (PrI), replay, and SP impersonation (SPI) attacks [5]. After accomplishing the authentication process, SM and SP need to create a common session key (SEK) to protect the communicated information. Secondly, the authentication protocol needs to guarantee the authenticity of the SM and SP, verify the data’s integrity, and ensure non-repudiation. Thirdly, by capturing an SM by an adversary, the procured sensitive information from the memory of the captured SM should not breach the security of the communication between other SMs and SP [6,7].

#### 1.1.2. Efficiency

In general, an SP has sufficient computational resources and can process a specific volume of information. However, many SMs communicate with SP concurrently in the SG system, requiring significant computational resources. Moreover, SMs are resource-limited devices with limited computational, communication, and energy resources. Thus, it is imperative to devise a resource-efficient authentication protocol that requires the least computational resources of SP and SM during the authentication process [4,8].

## 2. Related Work

Security and privacy are the critical parameters of concern for the SG systems. Various security schemes have been proposed to cope with the security challenges in the SG system [9,10]. Li et al. [4] proposed an AC mechanism, which is in-efficacious in thwarting replay, MIDM, and EPSL attacks. In addition, the proposed scheme is incapable of rendering MA and anonymity features. Kumar et al. [11] proposed an AC mechanism for the SG environment employing elliptic curve cryptography (ECC) and SHA. However, the scheme of Kumar et al. is incapable of restraining MIDM device impersonation. DIMP and EPSL attacks are unable to ensure mutual authentication (MA) and the security of SEK. An authentication protocol for the SG environment is presented in [12], using PUF and SHA. Similarly, a secure communication protocol for the SG environment is presented in [13], which is unable to withstand DoS and EPSL attacks. An ECC, XOR, and SHA-based lightweight AC protocol for the SG environment is presented in [14], which cannot withstand various security attacks. An authentication and SEK establishment scheme is propounded in [15], utilizing ECC, XOR, and SHA. The authors in [16] propounded a reliable AC protocol using ECC for the SG infrastructure that can hinder different security threats. In this paper, we propose a physical unclonable function (PUF)-based AC mechanism for the SG system. Li et al. [4] devised a pairing-based message AC protocol for the SG environment, unable to withstand the MIDM, DoS, EPSL, and impersonation attacks and incapable of providing security for SEK. Chen et al. [3] propounded a BP-based AC protocol for SG environments, incapable of resisting EPSL and impersonation attacks and incapable of ensuring the security of SEK. The security framework proposed in [17] cannot resist the DeS attack. An AE-based security framework is presented in [18], and its security is proved through the AVISPA. A detailed summary of various AC protocols or schemes propounded for the SG environment is presented in Table 1.

### 2.1. Motivation

Most of the AC protocols in the existing literature are devised using standardized symmetric encryption, such as AES, and public-key cryptography, such as ECC. These standardized cryptographic primitives are computationally expensive for resource-limited devices [14,26]. Moreover, most AC protocols are susceptible to various security risks, including DeS, replay, impersonation attacks, etc., as summarized in Section 2. Therefore, it is imperative to devise a secure and lightweight AC protocol for the SG systems.

Various AEAD schemes are devised to enable encryption and decryption services in resource-limited IoT devices. The main features of AEAD schemes are given to clarify why adopting the LWC primitives is essential when devising an AC protocol. This property of AEAD schemes makes them efficacious in reducing the encryption/decryption operations required to perform the AC process. (i) LWC-based AEAD schemes achieve message authenticity, integrity, and confidentiality simultaneously with a single encryption/decryption operation. (ii) AEAD schemes demand less computational and energy resources with reduced message overhead. (iii) The LWC-based hash function (Esch256) demands fewer computational resources than the existing hash functions while proffering the same security level.

**Figure 1:**
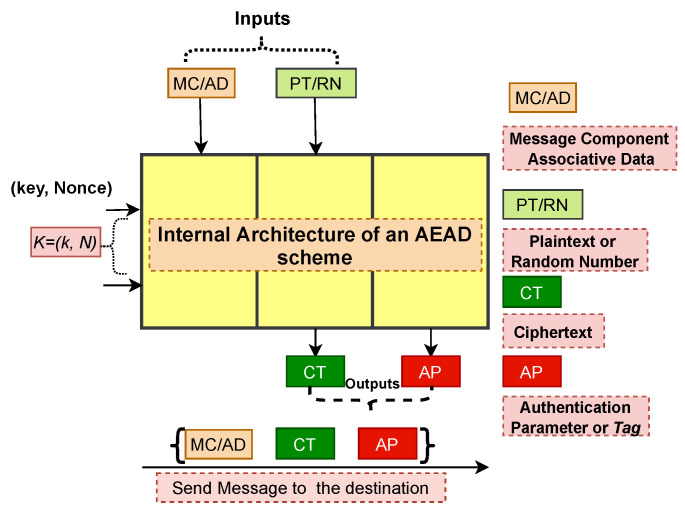
Message generation at source node using AEAD scheme.

Figure 1 presents the high-level working of an AEAD scheme, which is the base mechanism of the proposed AC protocol. Here, the AEAD scheme at the source node accepts the key along with associative data (AD), initialization vector/nonce, and plaintext as inputs to return output in the form of ciphertext (CT) and authentication parameters (AP). Moreover, the source generates a message with credentials {AD,CT,AP} and sends this message to the destination to accomplish MA. In the proposed protocol, AD comprises the temporary identity of the source node, i.e., AD={temporaryidentity,IPheader,etc.}. SP uses the temporary identity to find the record associated with the source from its memory. CT is obtained after encrypting the random numbers and other parameters used in the construction of SEK. At the destination, decryption is performed by using the AEAD scheme. The AEAD scheme generates the PT and $AP_{d}$ after taking the same input parameters as taken at the source node. To authenticate the validity of the obtained message, the destination node checks the condition $AP=AP_{d}$. If it holds, the received message is valid. We adopt the same methodology to propose a secure and lightweight AC protocol for the SG environment.

### 2.2. Research Contributions

The paper comprises the subsequent contributions.

1. This paper proffers a new lightweight AC protocol for SGs, called LACP-SG, which utilizes “Counter Mode Encryption with authentication Tag” (COMET) [27] along with a lightweight hash function “Esch256”. LACP-SG enables SP to check the authenticity of SM installed in the SG system before commencing the information exchange process. In addition, LACP-SG enables both the SM and SP to generate a shared SEK for future indecipherable communications.
2. The random oracle model (ROM) is utilized to corroborate the security of the established shared SEK. Moreover, security analysis utilizing the Scyther tool is executed to demonstrate that LACP-SG is resilient against MIDM, DeS, and replay attacks. Informal security is performed to illustrate that LACP-SG is resistant to SM capture and impersonation attacks. Moreover, LACP-SG allows the sensitive credentials associated with SM to be stored in ciphertext form in the database of SP, thereby restraining the PrI attack.
3. The meticulous comparative analysis is conducted to illustrate that LACP-SG renders enhanced security features while requiring low communication, storage, and computational overheads, respectively, than the related eminent AC protocols.

The subsequent paper is formed as follows. The system models, such as the network and attack model for LACP-SG, are illustrated in Section 3. Section 4 explicates the preliminary knowledge used in designing LACP-SG. The propounded LACP-SG is explicated in Section 5. The resiliency of LACP-SG against various attacks is furnished in Section 6. The significance of the LACP-SG is studied in Section 7. The paper concludes with concluding statements in Section 8.

## 3. System Model

### 3.1. Network Model

**Figure 2:**
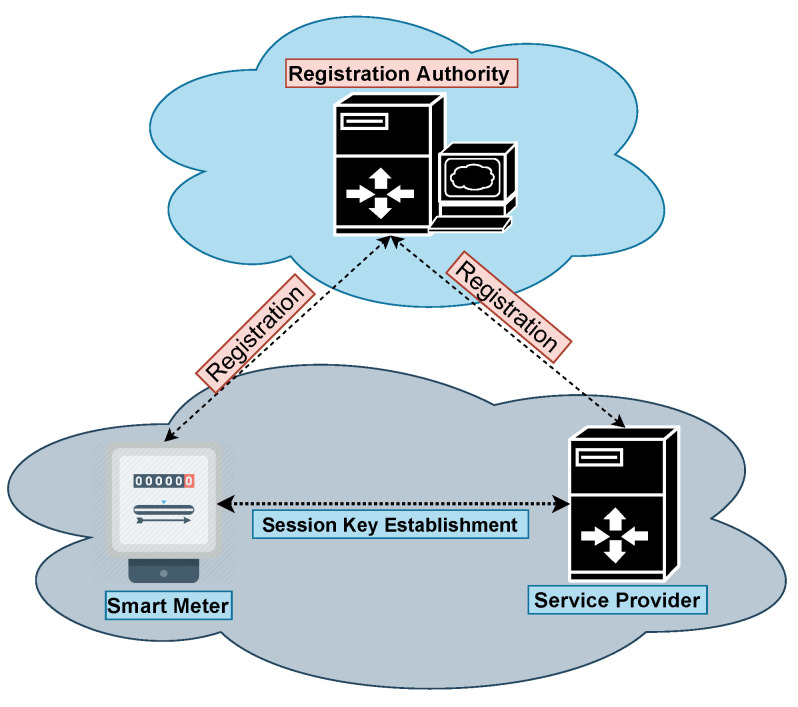
SG network.

For the authentication process, we contemplate the SG network model as depicted in Figure 2, which constitutes registration authority (RA), smart meter ($SM_{i}|i=1,2,\cdots ,n$), where “*n*” symbolizes the installed SMs and $\left(SP_{k}|k=1,2,\cdots ,N\right)$, where “*N*” symbolizes the number of SPs installed by RA. RA is liable for the registration of $SP_{k}$. $SP_{k}$ stores the data or information sent by $SM_{i}$. $SP_{k}$ pre-loads the confidential credentials into $SM_{i}^{\prime }s$ memory before its deployment in the SG environment. $SM_{i}$ collects the sensitive information and transmits the accumulated information to $SP_{k}$ via an openly available wireless channel, which is imperiled by different security vulnerabilities. Thus, ensuring the transmitted information’s integrity and confidentiality is inevitable. In the subsequent sections, the propounded secure AC protocol is elaborated, which validates the authenticity of the deployed $SM_{i}$. For encrypted communications, it sets up a secret key between $SP_{k}$ and $SM_{i}$.

### 3.2. Threat Model

We are considering the broadly utilized Dolev–Yao (DY) model for the proposed LACP-SG for the SG system [16,28]. The adversary A is able to alter and remove the content of the captured message. Furthermore, after updating the content of the captured message with malicious code, A can generate a malicious message. Network entities such as $SM_{i}$ can be physically compromised by A. Moreover, A can obtain sensitive data loaded in the memory of $SM_{i}$. In addition to this, A can use the procured information to carry out various attacks. In addition, $SP_{k}$ is contemplated as the trusted entity of the SG system. As in the DY model, in the CK-adversary model, A can not only intercept communications in the SG environment, but the secret parameters, such as session keys and state and private keys, can also be compromised by A.

## 4. Preliminaries

### 4.1. COMET

We use CHAM-based block cipher COMET-128 as the encryption/decryption scheme in the proposed LACP-SG. COMET is an AEAD scheme [27]. We express the encryption and decryption of COMET by (CTx, $AP_{tag}$) = $E_{K}${(N,AD),PTx} and (PTx, $AP_{tag}^{{}^{\prime }}$) = $D_{K}${(N,AD),CTx}, respectively, where *K*, *N*, AD, CTx, $AP_{tag}$, and PTx signifies “secret key”, “nonce”, “associative data”, “ciphertext”, “authentication parameter”, and “plaintext”, respectively. COMET decryption process will retrieve the plaintext if the condition $AP_{tag}^{}=AP_{tag}^{\prime }$ holds.

### 4.2. Esch256

We use the hash function “Esch256” in designing LACP-SG, which is faster than SHA-160/256 and requires fewer computational resources. In addition, Esch256 renders the same functionality as provided by SHA-160/256 with an output size of 256 bits. Moreover, Esch256 renders enhanced security features.

### 4.3. Physical Unclonable Function

(PUF) is a one-way function. PUF produces a unique output (response) after taking the challenge as the input parameter. The operation of PUF can be represented as R=PUF(CH).

### 4.4. Fuzzy Extractor

(FE) comprises two algorithms, namely, Generator Gen(·) and Reproducer Rep(·). The probabilistic algorithm Gen(·) produces key $K_{SM_{i}}$ and Helper Data (HD) by taking bio-metric *R* of user, i.e., $\left(K_{SM_{i}},HD\right)=Gen\left(R\right)$. Rep(·) is a deterministic algorithm that reproduce $K_{SM}$ by considering the inputs *R* and HD, if the condition $HM(R,R^{{}^{\prime }})\leq et$ holds, where HM is the hamming distance between *R* and $R^{\prime }$ and et is the error tolerance.

## 5. The Proposed LACP-SG Protocol

The proposed LACP-SG protocol comprises four phases: (1) SM deployment phase; (2) SP Deployment Phase; (3) AC Phase; and (4) New SM Deployment. The subsequent subsections explain the details of the designed LACP-SG protocol. It is assumed that all the participants in the SG environment are time-synchronized to cope with replay attacks. Table 2 lists the notations utilized in devising LACP-SG.

### 5.1. SP Deployment Phase

The SP deployment phase is accomplished by RA to deploy $SP_{k}$. For this, RA picks a unique identity $ID_{SP_{k}}$ and computes the secret key for the $SP_{k}$ deployed in SG environment as $K_{SP_{k}}=H\left(K_{RA}\|ID_{SP_{k}}\right)$, where $K_{RA}$ is the private key of RA. In addition, RA stores the list of credentials {$ID_{SP_{k}}$, $K_{SP_{k}}$} in the temper-resistance database of $SP_{k}$. RA also stores the credentials {$ID_{SP_{k}}$, $K_{SP_{k}}$} in its own database.

### 5.2. SM Deployment Phase

$SM_{i}$ deployment phase (SDP) is executed by RA. RA stores the secret credentials before $SM_{i}$ deployment in the SG environment by performing the trailing necessary steps.

#### 5.2.1. Step SDP-1

$SM_{i}$ picks a real identity $ID_{SM_{i}}$ of size 128 bits and a random number $RN_{r}$ of size 128 bits. $SM_{i}$ fabricates a message with parameters {$ID_{SM_{i}}$, $RN_{r}$} and sends it to RA through a secure channel. RA picks a challenge parameter $CH_{SM_{i}}$ and computes temporary identity $TID_{SM_{i}}$ = $(ID_{SM_{i}}$‖$RN_{SM_{i}})$ ⊕ $CP_{SP_{k}}$, where $CP_{SP_{k}}$ = $H(ID_{SP_{k}}$‖$K_{SP_{k}})$. In addition to this, RA computes $U=H(ID_{SM_{i}})$ and determines $SID_{i}$ = $(U_{1}$ ⊕ $U_{2})$, where $U_{1}$ and $U_{2}$ are derived by splitting *U* into two same-sized chunks, each with the size of 128 bits. RA sends the credentials {$CH_{SM_{i}}$, $TID_{SM_{i}}$} to $SM_{i}$ via the secure channel.

#### 5.2.2. Step SDP-2

After receiving the parameters {$CH_{SM_{i}}$, $TID_{SM_{i}}$} from RA, $SM_{i}$ generates a response by using PUF function as $R_{i}$ = $PUF(CH_{SM_{i}})$. In addition, $SM_{i}$ by using FE computes $(K_{SM_{i}}$, HD) = $Gen(R_{i})$ and sends $K_{SM_{i}}$ to $SP_{k}$ through a protected channel. Finally, $SM_{i}$ keeps the credentials {$TID_{SM_{i}}$, $CH_{SM_{i}}$, $RN_{r}$, HD} in its own memory.

#### 5.2.3. Step SDP-3

Upon obtaining $K_{SM_{i}}$ from $SM_{i}$, RA computes $B_{i}$ = ($K_{SM_{i}}$‖$RN_{r}$) ⊕ $CP_{SP_{k}}$. Finally, RA stores the parameters {$SID_{i}$, $B_{i}$} in the database of $SP_{k}$.

### 5.3. AC Phase

In AC phase (ACP), $SM_{i}$ achieves MA with $SP_{k}$. Moreover, $SM_{i}$ establishes a secret SEK with $SP_{k}$ to achieve encrypted communication. The trailing steps provide a detailed explanation of the AC phase.

#### 5.3.1. Step ACP-1

$SM_{i}$ retrieves $CH_{SM_{i}}$ from its memory, stored in the $SM_{i}$ memory during its deployment phase and computes $R_{i}=PUF\left(CH_{SM_{i}}\right)$. $SM_{i}$ regenerates $K_{SM_{i}}$ by using FE as $K_{SM_{i}}$ = $Rep(R_{i},HD)$, where the size of $K_{SM_{i}}$ is 128 bits. In addition, $SM_{i}$ selects the current timestamps $TS_{1}$ with size 32 bits, the random number $RN_{1}$ with size 128 bits, and computes $A_{}=H\left(TS_{1}\|RN_{r}\right)$ and nonce $N_{1}=A_{1}⊕A_{2}$, where $A_{1}$ and $A_{2}$ are procured by splitting *A* into two same-sized chunks, each with the size of 128 bits. In addition, $SM_{i}$ computes the associative data $AD_{1}=X_{1}⊕X_{2}$, where $X_{1}$ and $X_{2}$ are two equal parts of $TID_{SM_{i}}$. The size of $N_{1}$ and $AD_{1}$ is 128 bits. $SM_{i}$ by using COMET computes ($CT_{1},AP_{tag1}$) = $E_{K_{SM_{i}}}$$\{\left(N_{1},AD_{1}\right)$, $RN_{1}\}$, where $CT_{1}$, $AP_{tag1}$, and $RN_{1}$ denote ciphertext, authentication parameter (Tag), and plaintext, respectively. Finally, $SM_{i}$ constructs a message $M_{1}$: {$TS_{1}$, $TID_{SM_{i}}$, $CT_{1}$, $AP_{tag1}$} and sends $M_{1}$ to $SP_{k}$ through a public communication channel.

#### 5.3.2. Step ACP-2

Upon procuring $M_{1}$ form $SM_{i}$, $SP_{k}$ checks the condition $T_{dly}\geq \left|T_{mrc}-TS_{1}\right|$ to validate the $M_{1}$ freshness, where $T_{dly}$ is the allowed time delay, $T_{mr}$ is the $M_{1}$ received time, and $TS_{1}$ designates the $M_{1}$ generation time. If the condition holds, $SP_{k}$ considers $M_{1}$ as the authentic message and proceeds with the AC process. Otherwise, $SP_{k}$ discards $M_{1}$ and obstructs the AC process. $SP_{k}$ determines the common parameter $CP_{SP_{k}}$ as $CP_{SP_{k}}$ = $H(ID_{SP_{k}}$‖$K_{SP_{k}})$. Moreover, $SP_{k}$ retrieves $ID_{SM_{i}}$ and $RN_{SM_{i}}$ by computing $TID_{SM_{i}}$ ⊕ $CP_{SP_{k}}$ = ($ID_{SM_{i}}$‖$RN_{SM_{i}}$), where $TID_{SM_{i}}$ is received with $M_{1}$ and $CP_{SP_{k}}$ is computed at $SP_{k}$. Additionally, $SP_{k}$ picks the retrieved $ID_{SM_{i}}$ and computes $Q_{}=H\left(ID_{SM_{i}}\right)$ and $SID_{i}=Q_{1}⊕Q_{2}$, where $Q_{1}$ and $Q_{2}$ are two chunks of $Q_{}$ each of 128 bits. In addition, $SP_{k}$ checks if $SID_{i}$ is located in its database (memory). If $SID_{i}$ is found, $SP_{k}$ retrieves the credential {$B_{i}$} corresponding to $SID_{i}$, stored in the database (memory) of $SP_{k}$. In addition to this, $SP_{k}$ computes $CP_{SP_{k}}$ ⊕ $B_{i}$ = $(RN_{r}$‖$K_{SM_{i}})$. Additionally, $SP_{k}$ determines $AA_{}$ = $H(TS_{1}$‖$RN_{r})$ and nonce $N_{2}$ = $AA_{1}⊕AA_{2}$, where $AA_{1}$ and $AA_{2}$ are procured by splitting AA into two same-sized chunks, each with the size of 128 bits. Furthermore, $SM_{i}$ computes $AD_{2}$ = $X_{1}^{a}$ ⊕ $X_{2}^{a}$, where $X_{1}^{a}$ and $X_{2}^{a}$ are two equal parts of $TID_{SM_{i}}$. Finally, $SP_{k}$ by using COMET computes ($RN_{1}$, $AP_{tag2}$) = $D_{K_{SM_{i}}}$$\{\left(N_{2},AD_{2}\right)$, $CT_{1}\}$, where $AD_{2}$, $N_{2}$, $CT_{1}$, $AP_{tag2}$, and $RN_{1}$ denote associative data, nonce, ciphertext, authentication parameter (Tag), and plaintext, respectively. To validate the authenticity of $M_{1}$, $SP_{k}$ checks the condition $AP_{tag1}=AP_{tag2}$. If it holds, $SP_{k}$ considers $M_{1}$ as the authentic message, which is received from a valid $SM_{i}$. Otherwise, $SP_{k}$ discards $M_{1}$ and aborts the AC process.

#### 5.3.3. Step ACP-3

After substantiating the authenticity of $M_{1}$, $SP_{k}$ picks timestamp $TS_{2}$, $RN_{2}$, $RN_{SM_{i}}^{n}$, and computes the new temporary identity $TID_{SM_{i}}^{new}$ as ($ID_{SM_{i}}$‖$RN_{SM_{i}}^{n}$) ⊕ $CP_{SP_{k}}$ = $TID_{SM_{i}}^{new}$, where $ID_{SM_{i}}$ is real identity of $SM_{i}$ and $RN_{SM_{i}}^{n}$ is a new random number. Moreover, $SP_{k}$ computes $K_{SM_{i}}^{1}$ = ($K_{SM_{i}}$ ⊕ $RN_{1}$), which is used in the encryption process. For encrypted communication in future, $SP_{k}$ computes SEK as $SK_{SP_{k}}$ = $H(TID_{SM_{i}}$‖$RN_{1}$‖$RN_{2}$ ⊕ $ID_{SM_{i}}$‖$TS_{2}$‖$TID_{SM_{i}}^{new})$ and calculates $SK_{v1}$ = $SK_{SP_{k}}^{a}$ ⊕ $SK_{SP_{k}}^{b}$. Furthermore, $SP_{k}$ determines $N_{3}=\left(RN_{r}⊕RN_{1}\right)$, and $PT_{1}=(TID_{SM_{i}}^{new}\|\left(RN_{2}⊕ID_{SM_{i}}\right)\left\|SK_{v1}\right)$. In addition to this, by using COMET, $SP_{k}$ computes ($CT_{2}$, $AP_{tag3}$) = $E_{K_{SM_{i}}^{1}}$$\{\left(N_{3},AD_{2}\right)$, $PT_{1}\}$, where $AD_{2}$, $N_{3}$, $CT_{2}$, $AP_{tag3}$, and $PT_{1}$ denote associative data, nonce, ciphertext, authentication parameter, and plaintext, respectively. Finally, $SP_{k}$ contrives a message $M_{2}$: {$TS_{2}$, $CT_{2}$, $AP_{tag3}$} and dispatches $M_{2}$ to $SM_{i}$ via an open/wireless channel.

#### 5.3.4. Step ACP-4

**Figure 3:**
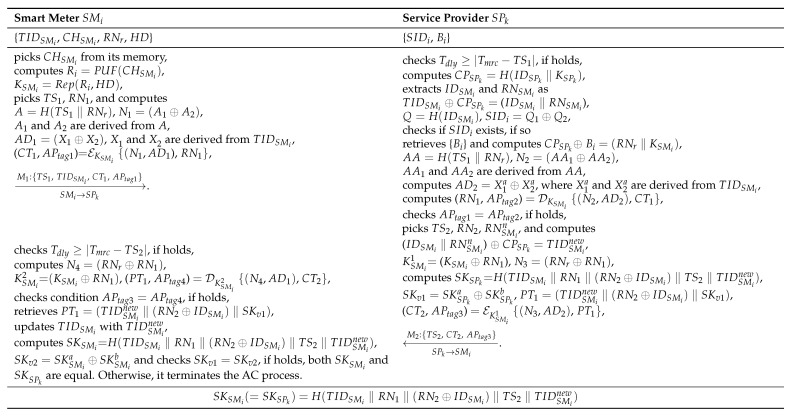
LACP-SG authentication phase.

After acquiring $M_{2}$ from $SP_{k}$, $SM_{i}$ checks the condition $T_{dly}\geq \left|T_{mrc}-TS_{2}\right|$ to validate the freshness of $M_{2}$. If $M_{2}$ is fresh, $SM_{i}$ determines $N_{4}=\left(RN_{r}⊕RN_{1}\right)$, $K_{SM_{i}}^{2}$ = $\left(K_{SM_{i}}⊕RN_{1}\right)$, and by using COMET computes ($PT_{1}$, $AP_{tag4}$)= $D_{K_{SM_{i}}^{2}}$$\{\left(N_{4},AD_{1}\right)$, $CT_{2}\}$, where $AD_{1}$, $N_{4}$, $CT_{2}$, and $AP_{tag4}$ denote associative data, nonce, ciphertext, authentication parameter (Tag), and plaintext, respectively. Moreover, $SM_{i}$ checks the condition $AP_{tag3}=AP_{tag4}$. If it holds, $SM_{i}$ procures the plaintext $PT_{1}=(TID_{SM_{i}}^{new}\|\left(RN_{2}⊕ID_{SM_{i}}\right)\left\|SK_{v1}\right)$ from the decryption process. For indecipherable communication, $SM_{i}$ computes the SEK as $SK_{SM_{i}}$ = $H(TID_{SM_{i}}$‖$RN_{1}$‖$RN_{2}$ ⊕ $ID_{SM_{i}}$‖$TS_{2}$‖$TID_{SM_{i}}^{new})$. In addition to this, $SM_{i}$ calculates $SK_{v2}$ = $SK_{SM_{i}}^{a}$ ⊕ $SK_{SM_{i}}^{b}$ and checks the condition $SK_{v1}=SK_{v2}$. If it holds, both $SK_{SM_{i}}^{}$ and $SK_{SP_{k}}^{}$ are equal. Otherwise, it terminates the AC process. Finally, $SM_{i}$ updates $TID_{SM_{i}}$ with $TID_{SM_{i}}^{new}$ in its own memory. Figure 3 summarizes the LACP-SG AC phase.

### 5.4. New SM Deployment Phase

RA performs the subsequent steps to deploy a new $SM_{i}^{n}$.

#### 5.4.1. Step SDP-1

$SM_{i}^{n}$ picks a real identity $ID_{SM_{i}}^{n}$ and $RN_{r}^{n}$ and sends {$ID_{SM_{i}}^{n}$, $RN_{r}^{n}$} to RA through a protected channel. RA picks a new challenge $CH_{SM_{i}}^{n}$ and computes the new temporary identity $TID_{SM_{i}}^{n}$ = $(ID_{SM_{i}}^{n}$‖$RN_{SM_{i}}^{n})$ ⊕ $CP_{SP_{k}}$. Moreover, RA computes $U^{n}=H\left(ID_{SM_{i}}^{n}\right)$ and derives $SID_{i}^{n}=\left(U_{1}^{n}⊕U_{2}^{n}\right)$, where $U_{1}^{n}$ and $U_{2}^{n}$ are derived by splitting $U^{n}$ into two same-sized chunks, each with the size 128 bits. RA sends the credentials {$CH_{SM_{i}}^{n}$, $TID_{SM_{i}}^{n}$} to $SM_{i}^{n}$ via a secure channel.

#### 5.4.2. Step SDP-2

After receiving a challenge $CH_{SM_{i}}^{n}$ from RA, $SM_{i}^{n}$ generates a response by using the PUF function as $R_{i}^{n}=PUF\left(CH_{SM_{i}}^{n}\right)$. In addition, $SM_{i}^{n}$ by using FE computes $(K_{SM_{i}}^{n}$, $HD^{n})$ = $Gen(R_{i}^{n})$ and sends $K_{SM_{i}}^{n}$ to RA via secure channel. Furthermore, $SM_{i}$ stores {$TID_{SM_{i}}^{n}$, $CH_{SM_{i}}^{n}$, $RN_{r}^{n}$} in its own memory. Upon receiving $K_{SM_{i}}^{n}$ from $SM_{i}^{n}$, RA computes. In addition, $SP_{k}$ computes $B_{i}^{n}$ = ($K_{SM_{i}}^{n}$‖$RN_{r}^{n}$) ⊕ $CP_{SP_{k}}$. Finally, RA stores the parameters {$SID_{i}^{n}$, $B_{i}^{n}$} in the $SP_{k}$ database.

## 6. Security Analysis

### 6.1. Informal Security Analysis

#### 6.1.1. Anonymity and Untraceability

Assume A eavesdrops the communicated messages, such as $M_{1}$: {$TS_{1}$, $TID_{SM_{i}}$, $CT_{1}$, $AP_{tag1}$} and $M_{2}$: {$TS_{2}$, $CT_{2}$, $AP_{tag3}$}, which are exchanged during the AC phase of the proposed LACP-SG. A cannot determine the real identity of SM of SP, which are $ID_{SM_{i}}$ and $ID_{SP_{k}}$, respectively, from the captured $M_{1}$ and $M_{2}$. A by capturing $M_{1}$ and $M_{2}$ cannot procure the real identities of SM and SP.

#### 6.1.2. Replay Attack

A after expropriating all the messages, such as $M_{1}$: {$TS_{1}$, $TID_{SM_{i}}$, $CT_{1}$, $AP_{tag1}$} and $M_{2}$: {$TS_{2}$, $CT_{2}$, $AP_{tag3}$} tries to regenerate the captured messages to obtain helpful information from the participants of the AC phase. However, we assume the system is time-synchronized, and each message bears the newest timestamp and random numbers. A cannot frame the replay attack because the entities $SM_{i}$ and $SP_{k}$ verify the newness/oldness of the obtained message by confirming the condition $T_{dly}\geq \left|T_{mrc}-TS_{1}\right|$ and $T_{dly}\geq \left|T_{mrc}-TS_{2}\right|$, respectively. If the obtained transmission is delayed, the entity of the receiving will dump the obtained message. In this way, the proposed LACP-SG detects the replayed messages and discards such received messages. Hence, LACP-SG is protected against replay attacks.

#### 6.1.3. DeS Attack

The proposed LACP-SG renders resistance against DeS attack. For anonymous communication, $SM_{i}$ uses $TID_{SM_{i}}$, which is updated by $SP_{k}$ during the accomplishment of every new AC session. $SP_{k}$ constructs $TID_{SM_{i}}$ by concatenating $ID_{SM_{i}}$ and a fresh random number $RN_{SM_{i}}$, i.e., $\left(ID_{SM_{i}}\|RN_{SM_{i}}\right)⊕CP_{SP_{k}}$, where $ID_{SM_{i}}$ remains constant and $RN_{SM_{i}}$ is updated to $RN_{SM_{i}}^{n}$. Suppose A drops $M_{2}$ during the execution of the AC phase. This action of A cannot affect the execution of the new AC session because $ID_{SM_{i}}$ is constant, which is extracted by $SP_{k}$ to compute the $SID_{i}$. $SID_{i}$ is used to find the record at $SP_{k}$ related to $SM_{i}$. So, LACP-SG is capable of resisting the DeS attack.

#### 6.1.4. Privilege Insider Attack

To accomplish the authentication phase in the proposed LACP-SG scheme, $SP_{k}$ stores the parameters {$SID_{i}$, $B_{i}$} in the database. Thus, to fabricate a valid messages, such as $M_{1}$: {$TS_{1}$, $TID_{SM_{i}}$, $CT_{1}$, $AP_{tag1}$} and $M_{2}$: {$TS_{2}$, $CT_{2}$, $AP_{tag3}$}, it is imperative for A to compute $CP_{SP_{k}}$ ⊕ $B_{i}$ = $(RN_{r}$‖$K_{SM_{i}})$. However, without knowing the secret key of $SP_{k}$, it is hard for A to extract $RN_{r}$ and $K_{SM_{i}}$, which are required to construct $M_{1}$ and $M_{2}$. Hence, LACP-SG can resist the PrI attack.

#### 6.1.5. MIDM Attack

Assume that A expropriates all the exchanged messages $M_{1}$ and $M_{2}$ between the entities during the AC phase over the wireless/open communication channel. Now, A may attempt to reconstruct the seized messages to make the participants of the system believe that the received messages are generated by licit entities. To simulate a licit message $M_{1}$ on behalf of $SM_{i}$, A requires to have all the confidential/secret credentials of $SM_{i}$, i.e., {$ID_{SM_{i}}$, $CH_{i}$, $K_{SM_{i}}$}. Similarly, A needs to extricate all the secret/confidential parameters of $SP_{k}$ to construct a valid response message on behalf of $SP_{k}$. However, without having all the confidential credentials of $SM_{i}$ and $SP_{k}$, it is impractical for A to construct a valid message. Therefore, LACP-SG can restrain MIDM attacks.

#### 6.1.6. Impersonation/Modification/Injection Attack

To impersonate as $SP_{k}$, A has to regenerate the message $M_{2}$ on behalf of $SP_{k}$ to make $SM_{i}$ believe that the message is licit and obtained from an honest $SP_{k}$. Now, suppose A attempts to generate $M_{1}$ with valid credentials. However, to generate $M_{2}$, A requires knowing the confidential credentials of $SP_{k}$. However, A cannot produce a valid message $M_{2}$ in polynomial time without knowing the secret credentials to emulate as legitimate $SP_{k}$. Similarly, A requires knowing the confidential credentials of $SM_{i}$. Therefore, LACP-SG is protected against $SM_{i}$ and $SP_{k}$ impersonation attacks.

#### 6.1.7. Key Compromise Impersonation Attack

In this attack, A tries to impersonate as a valid $SM_{i}$ by compromising the long-term secret key of $SP_{k}$. However, to construct a valid message $M_{1}$: {$TS_{1}$, $TID_{SM_{i}}$, $CT_{1}$, $AP_{tag1}$}, it is necessary for A to obtain the secret parameters, such as $RN_{r}$ and $K_{SM_{i}}$. Thus, without having these confidential parameters, it is hard for A to impersonate a valid $SM_{i}$. Similarly, without having the confidential parameters of $SP_{k}$, A cannot impersonate a licit $SP_{k}$. In this way, LACP-SG can resist key compromise impersonation attacks.

#### 6.1.8. Known Session-Specific Temporary Information Leakage/EPSL Attack

According to the CK-adversary model, A can compromise the secret credentials (Long Term Secrets (LTS), Ephemeral Secrets (ES)), and session states aside from all the actions allowed under the DY model. In LACP-SG, the session key is created using both LTS and ES, i.e., $SK_{SM_{i}}\left(=SK_{SP_{k}}\right)$ = $H(TID_{SM_{i}}\|RN_{1}\|\left(RN_{2}⊕ID_{SM_{i}}\right)\|TS_{2}\|TID_{SM_{i}}^{new})$. Therefore, it is imperative for A to guess that both LTS and ES construct the session key.

#### 6.1.9. SM Capture/Memory Modification Attack

According to the DY threat model, A can seize some of the SMs from in the SG environment. A can extricate the secret credentials by using a power analysis attack kept in the memory of SM. However, the parameters $CH_{i}$, $RN_{r}$, and $TID_{SM_{i}}$ are unlike for all SMs installed in the SG environment. Therefore, by capturing some of the installed SMs, A cannot compromise the security of the whole SG environment. Hence, LACP-SG is resilient against SM capture attacks.

### 6.2. ROM-Based Formal Security Analysis

This section provides a ROM-based analysis of the SEK security between $SM_{i}$ and $SP_{k}$ during the execution of the AC phase of LACP-SG. The subsequent components are described in the ROM model.

**Participants:** Suppose that $\Psi_{RA}^{t1}$, $\Psi_{SM_{i}}^{t2}$, and $\Psi_{SP_{k}}^{t3}$ represent instances t1, t2, and t3 of the participants RA, $SM_{i}$, and $SP_{k}$, denoted as oracles.

**Accepted** state: When an instance $\Psi^{t}$ acquires the last message, it will be in the accepted state. The session identification (Sid) of $\Psi^{t}$ for the current session prescribes the ordered sequence of all exchanged messages (i.e., messages sent/received by $\Psi^{t}$).

**Partnering:** Two instances $\Psi^{t2}$ and $\Psi^{t2}$ are partners only if both are in an acceptable state and share similar session keys.

**Freshness:**A is unable to obtain the SEK established between $SM_{i}$ and $SP_{k}$ by running the Reveal query presented in Table 3.

**Adversary:**A can fully control and seize all the messages and alter, falsify, and infiltrate messages by employing the queries expressed in Table 3. A can execute the hash function H(.), referred to as random oracle ESHah.

**Definition** **1.** *Online chosen ciphertext attack (OCCA3) advantage of A, which is executing against an AEAD scheme in polynomial-time (pt), can be defined as follows.*

(1) $$\begin{array}{c} Adv_{\varphi }^{OCCA3}\left(A\right)\leq Adv_{\varphi }^{OPRP-CPA}\left(que,len,pt\right)\\ +Adv_{\varphi }^{INT-CTXT}\left(que,len,pt\right), \end{array}$$

**Theorem** **1.** *Let A run against LACP-SG in pt to derive the established SEK between $SM_{i}$ and $SP_{k}$ during the AC phase. Let $H_{que}$ signify Esch256 queries, |ESHah| designates the range space of Esch256 output, $H_{puf}$ represents PUF quires, |PUF| designates the range space of PUF output, and $Adv_{COMET,A}^{OCCA3}\left(que,len,pt\right)$ is the advantage in compromising the security of an online AEAD scheme (COMET) (Definition 1). The maximum advantage of A for compromising the security of SEK, established between $SM_{i}$ and $SP_{k}$, can be described as follows:*

(2) $$\begin{array}{c} Adv_{A}^{LACP-SG}\left(pt\right)\leq \frac{H_{que}^{2}}{\left|ESHah\right|}+\frac{H_{puf}^{2}}{\left|PUF\right|}\\ +2.Adv_{COMET,A}^{OCCA3}\left(que,len,pt\right). \end{array}$$

**Proof.**  The succeeding five games $\left(GM_{z}|z=0,1,2,3,4\right)$ are executed to prove Theorem 1. We heed the identical means to establish the proof of Theorem 1 as followed in [29,30,31,32,33]. In addition to this, we characterize the A advantage in compromising the security of SEK by $Adv_{A}^{LACP-SG}\left(pt\right)$ = |2·Pr[SuS]−1|, where “Pr[SuS]” indicates the possibility of a circumstance where A can achieve/win the game. LACP-SG is defended if $Adv_{A}^{LACP-SG}$(pt) is insignificant.

$GAM_{0}$: In this game, A performs an active attack against LACP-SG under ROM. A at the commencement of $GM_{0}$ guesses the bit $C^{\prime }$ randomly. Then, trailing can be achieved

(3) $$Adv_{A}^{LACP-SG}\left(pt\right)=\left|2.Pr\left[SuS0\right]-1\right|.$$

$GAM_{1}$: In $GAM_{1}$, A makes the execute query to effectuate the eavesdrop attack. By effectuating eavesdrop attack during the execution of AC phase, A can intercept all the exchanged messages, such as $M_{1}$: {$TS_{1}$, $TID_{SM_{i}}$, $CT_{1}$, $AP_{tag1}$} and $M_{2}$: {$TS_{2}$, $CT_{2}$, $AP_{tag3}$}. A effectuates Test at the end of this game and validates whether the outcome of the Test query is a random number or a real session key, i.e., $SK_{SM_{i}}\left(=SK_{SP_{k}}\right)$ = $H(TID_{SM_{i}}\|RN_{1}\|\left(RN_{2}⊕ID_{SM_{i}}\right)\|TS_{2}\|TID_{SM_{i}}^{new})$, where $TID_{SM_{i}}^{new}=\left(ID_{SM_{i}}\|RN\right)⊕CP_{SP_{k}}$. The session key is produced in the proposed LACP-SG using the LTS and ES. Therefore, to reveal the session key established between $SM_{i}$ and $SP_{k}$, it is imperative for A to guess both the ES and LTS simultaneously. However, it is impractical for A to procure all the secret parameters by capturing $M_{1}$ and $M_{2}$. So, the winning chance of this game for A will not increase by effectuating the eavesdrop attack:

(4) Pr[SuS0]=Pr[SuS1].

$GAM_{2}$: In this game, the aim of A is to deceive an entity to receive a mutated message. A is authorized to make various ESHah queries to check the presence of the hash collisions. All the exchanged messages, such as $M_{1}$: {$TS_{1}$, $TID_{SM_{i}}$, $CT_{1}$, $AP_{tag1}$} and $M_{2}$: {$TS_{2}$, $CT_{2}$, $AP_{tag3}$} during the AC phase indirectly include the associative data and nonce, and temporary identities, which are protected by the collision-resistant Esch256 hash function. Therefore, there will be no collision when A performs Send queries. The consequences of the birthday paradox confer

(5) $$\left|Pr\left[SuS1\right]-Pr\left[SuS2\right]\right|\leq \frac{H_{que}^{2}}{2|ESHah|}.$$

$GAM_{3}$: This game is considered a continuation of $GAM_{2}$ that simulates PUF queries. According to $GAM_{2}$, it follows that

(6) $$\left|Pr\left[SuS3\right]-Pr\left[SuS2\right]\right|\leq \frac{H_{puf}^{2}}{2|PUF|}.$$

$GAM_{4}$: In this game, A attempts to construct the session key by capturing $M_{1}$ and $M_{2}$, which are protected by AEAD scheme. In LACP-SG the session key in constructed as $SK_{SM_{i}}\left(=SK_{SP_{k}}\right)$ = $H(TID_{SM_{i}}\|RN_{1}\|\left(RN_{2}⊕ID_{SM_{i}}\right)\|TS_{2}\|TID_{SM_{i}}^{new})$. Therefore, A has to procure $RN_{1}$ and $RN_{2}$, which are encrypted using AEAD scheme (COMET). Moreover, the associative data and the initialization vector used in the encryption process are random. In addition, secret keys are required to decrypt $CT_{1}$ and $CT_{2}$. It is computationally impractical to perform the decryption process in polynomial time. Due to OCCA3 property (Definition 1), it then follows that

(7) $$\begin{array}{c} \left|Pr\left[SuS3\right]-Pr\left[SuS4\right]\right|\leq Adv_{COMET,A}^{OCCA3}\left(que,len,pt\right). \end{array}$$

As all the queries are performed, A executes the Test queries to presume bit $C^{\prime }$ for winning the game. Thus, we obtain

(8) Pr[SuS4]=1/2.

From (3) and (4), we obtain

(9) $$Adv_{A}^{LACP-SG}\left(pt\right)=\left|2.Pr\left[SuS0\right]-\frac{1}{2}\right|.$$

From (9), we obtain

(10) $$\frac{1}{2}.Adv_{A}^{LACP-SG}\left(pt\right)=\left|Pr\left[SuS0\right]-\frac{1}{2}\right|.$$

By using (8) and (10), we obtain

(11) $$\frac{1}{2}.Adv_{A}^{LACP-SG}\left(pt\right)=\left|Pr\left[SuS1\right]-Pr\left[SuS4\right]\right|$$

Through triangular inequality, we obtain

(12) $$\begin{array}{c} \left|Pr[SuS1]-Pr[SuS4]|\leq |Pr[SuS1]-Pr[SuS2]\right|\\ +|Pr[SuS2]-Pr[SuS4]|\\ \leq |Pr[SuS1]-Pr[SuS2]|+|Pr[SuS2]-Pr[SuS3]|\\ +|Pr[SuS3]-Pr[SuS4]|. \end{array}$$

By utilizing (5), (6), (7) and (12), we obtain

(13) $$\begin{array}{c} Adv_{A}^{LACP-SG}\left(pt\right)\leq \frac{H_{que}^{2}}{\left|ESHah\right|}+\frac{H_{puf}^{2}}{\left|PUF\right|}\\ +2.Adv_{COMET,A}^{OCCA3}\left(que,len,pt\right). \end{array}$$

□

### 6.3. Scyther Based Formal Security Verification

**Figure 4:**
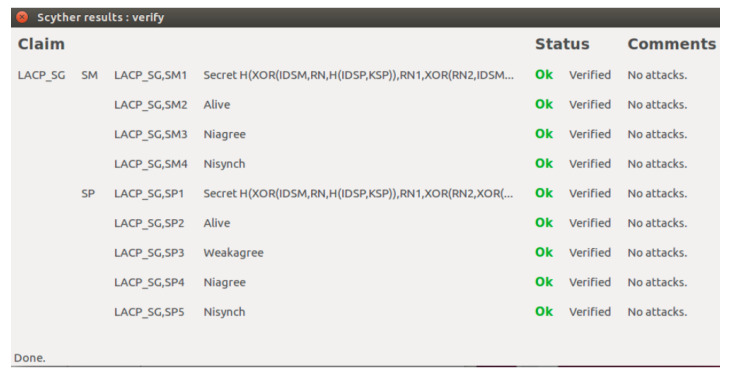
Security analysis of LACP-SG using Scyther.

We investigated the formal security of LACP-SG by utilizing the widely adopted validation tools, i.e., Scyther. Scyther is a Python-based software designed to formally analyze the security of the authentication schemes, their security claims, and potential vulnerabilities. Scyther employs the Security Protocol Description Language (SPDL) for describing a devised security scheme and is also utilized to determine the weaknesses of a security scheme by demonstrating any potential threats or risks. In the proposed LACP-SG, two roles are defined, such as $SM_{i}$ and $SP_{k}$. There are two manually specified claims, such as claim(SM,Secret,SK) and claim(SP,Secret,SK), which are validated by Scyther, as shown in Figure 4. In addition, Scyther also generates the claims, such as claim(SM,Alive), claim(SM,Nisynch), and claim(SM,Niagree), which are validated as demonstrated in Figure 4.

## 7. Performance Evaluation

LACP-SG is contrasted with other protocols, such as in Bera et al. [29], Chaudhry et al. [30], Bera et al. [34], Kumar et al. [11], Chaudhry et al. [35], and Mehmood et al. [20]. We use the Python-based library “PyCrypto” along with COMET code to acquire the time complexity of cryptographic primitives and COMET. Table 4 depicts the time complexities of different cryptographic operations.

### 7.1. Security Comparison

A comparison of the security properties of LACP-SG and other related AC schemes is demonstrated in Table 5. That of Bera et al. [29] cannot restrain the DeS attack, that of Bera et al. [34] is unprotected against the DeS attack, and that of Mehmood et al. [20] is insecure against the DoS, MIDM, PrI, EPSL, RA attacks and does not provide the SEK security. The scheme of Kumar et al. [11] is against DIMP, MIDM, and EPSL attacks and does not provide SEK security. In addition to this, the scheme of Chaudhry et al. [35] is incapable of resisting EPSL, SIMP, DIMP, device capture, and SEK disclosure attacks. Moreover, Chaudhry et al. [30] provide insecure certificate computation, which causes various attacks, such as device capture and DIMP attacks. However, the proposed LACP-SG is secure and protected against various pernicious attacks, such as MIDM and DeS attacks.

### 7.2. Communication Overhead Comparison

**Figure 5:**
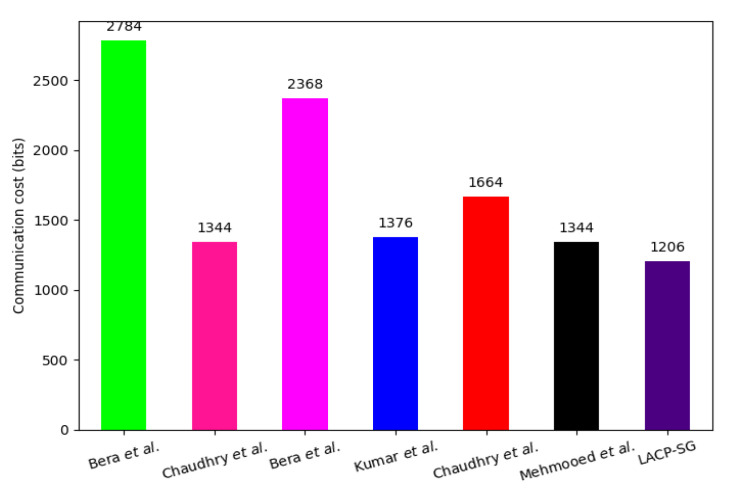
Communication cost needed to perform the AC phase (single $SM_{i}$) [11,20,29,30,34,35].

For analyzing the communication overhead that occurred during the AC phase, we suppose that the length of the ECC point, identity, hash function output, initialization vector/random number/nonce, and timestamp are 320, 128, 256, 128, and 32 bits, respectively. There are two messages required to accomplish the AC phase of LACP-SG, i.e., $M_{1}$: {$TS_{1}$, $TID_{SM_{i}}$, $CT_{1}$, $AP_{tag1}$}, $M_{2}$: {$TS_{2}$, $CT_{2}$, $AP_{tag3}$}. The sizes of $M_{1}$ and $M_{2}$ are {32 + 256 + 128 + 128} = 544 bits and {32 + 512 + 128} = 662 bits. Hence, the communication cost of LACP-SG is {662 + 544} = 1206 bits, which is 56.68%, 10.27%, 49.07%, 12.35%, 27.52%, and 10.27% lesser than the scheme of Bera et al. [29], Chaudhry et al. [30], Bera et al. [34], Kumar et al. [11], Chaudhry et al. [35], and Mehmood et al. [20], respectively. The comparison between LACP-SG and the related AC protocol communication overhead is given in Table 6 and Figure 5.

### 7.3. Computational Overhead Comparison

**Figure 6:**
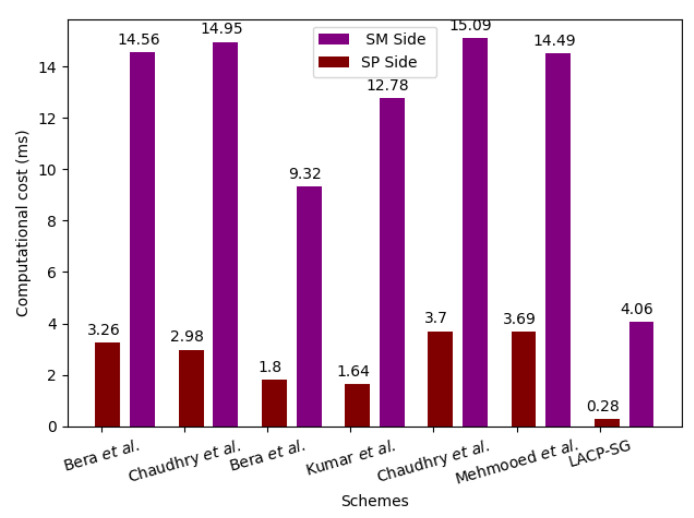
Computational cost at $SM_{i}$ and $SP_{k}$ side [11,20,29,30,34,35].

**Figure 7:**
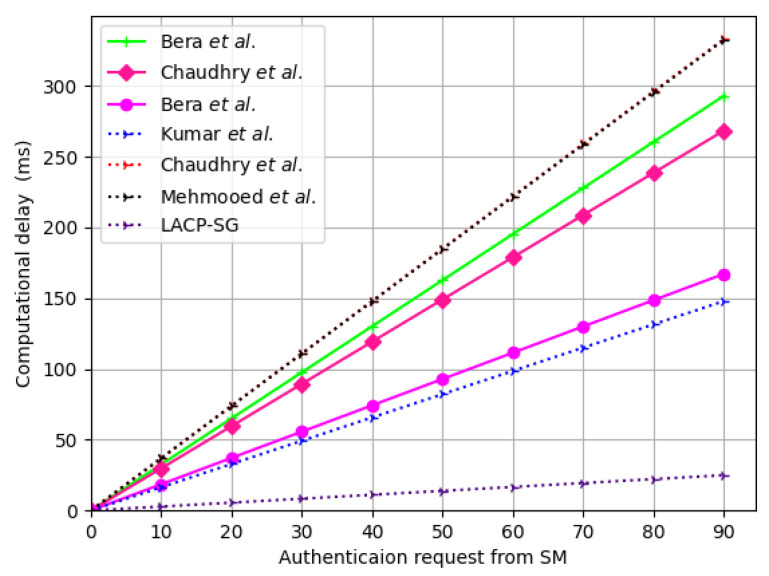
The computational cost increases with the number of authentication requests [11,20,29,30,34,35].

We employ the time complexity of different cryptographic operations, shown in Table 4, to estimate the computational overhead of LACP-SG and relevant AC protocol. LACP-SG requires the computational overhead of $7T_{HE}+4T_{co}+T_{rep}+T_{pu}\approx 4.34$ ms in the AC phase. The schemes of Bera et al. [29], Chaudhry et al. [30], Bera et al. [34], Mehmood et al. [20], Kumar et al. [11], and Chaudhry et al. [35] require $22T_{H}+8T_{ecc}+2T_{eca}\approx 17.82$ ms, $8T_{H}+9T_{ecc}+2T_{eca}\approx 17.93$ ms, $18T_{H}+4T_{en}+4T_{ecc}+2T_{eca}\approx 11.12$ ms, $12T_{H}+4T_{ecc}\approx 14.42$ ms, $8T_{H}+10T_{ecc}+4T_{eca}\approx 18.79$ ms, and $8T_{H}+9T_{ecc}+5T_{eca}\approx 18.18$ ms, respectively, which are 75.14%, 75.29%, 60.16%, 69.28%, 76.42%, and 75.63% higher than the proposed LACP-SG, respectively, as shown in Table 7. Moreover, the computational cost needed at the $SP_{k}$ and $SM_{i}$ side is shown in Figure 6, where it is obvious that LACP-SG incurs lesser computational cost than the related AC protocols. Furthermore, Figure 7 illustrates the comparison of the computational cost at $SP_{k}$ with increasing the authentication requests, which are generated by $SM_{i}$ in the SG environment.

### 7.4. Storage Overhead Comparison

**Figure 8:**
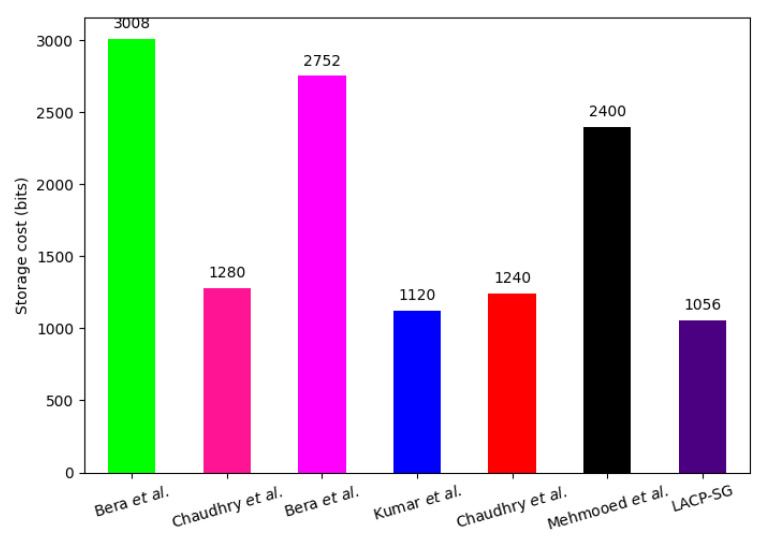
Total storage cost comparison [11,20,29,30,34,35].

In LACP-SG, the smart meter $SM_{i}$ and $SP_{k}$ requires storing {$CH_{SM_{i}}$, $TID_{SM_{i}}$, $RN_{r}$, HD} and {$SID_{i}$, $B_{i}$, $RN_{r}$} size of { 256 + 256 + 160} = 672 bits and {128 + 256 } = 384 bits. To execute the AC phase, the aggregated storage overhead of LACP-SG is {672 + 384} = 1056 bits. The schemes of Bera et al. [29], Chaudhry et al. [30], Bera et al. [34], Mehmood et al. [20], Kumar et al. [11], and Chaudhry et al. [35] require storing 3008 bits, 1280 bits, 2752 bits, 1120 bits, 1240 bits, and 2400 bits, respectively, which are 64.89%, 17.5%, 61.63%, 5.71%, 14.84%, 56%, 37.26% higher than the proposed LACP-SG, respectively. The comparison of LACP-SG and the related AC protocols’ storage overhead is given in Figure 8.

## 8. Conclusions

This paper presents an AC protocol called LACP-SG, which enables secure communication in the resource-constrained SG environment. To this end, LACP-SG validates the authenticity of the deployed SM and establishes a SEK between the SM and server to accomplish secure communications. The security of the established SEK is validated through ROM-based analysis. Moreover, through Scyther-based analysis, LACP-SG is found to be secure against MIDM and replay attacks. Informal security analysis reveals that the protocol is protected against de-synchronization and SM capture attacks. Finally, a rigorous comparative analysis shows that LACP-SG renders superior security and requires lower computational, storage, and communication cost than the related AC protocols, thereby advocating the feasibility of LACP-SG for SG applications. 

## Figures and Tables

**Table 1:** Summary of various AC protocols.

AC/AKE Protocol	Shortcomings/Security Vulnerabilities
Wu et al. [19]	Unable to thwart MIDM and EPSL attacks. Incapable of rendering anonymity and PFS features.
Mahmood et al. [20]	In-efficacious in preventing DoS, impersonation, PrI, replay, MIDM, and EPSL attacks.
Dariush et al. [21]	In-efficacious in resisting DoS attack. Incapable of rendering SM’s anonymity and SEK security.
Banerjee et al. [22]	Unable to render identity protection and traceability.
Wazid et al. [23]	Exposed to DeS attack. Incapable of rendering revocability and formal validation.
Odelu et al. [24]	In-efficacious in preventing DoS, MIDM, and impersonation attacks. Unable to assure SM’s anonymity.
Xie et al. [25]	In-efficacious in resisting replay and impersonation attacks. Incapable of rendering forward secrecy.
Li et al. [4]	In-efficacious in thwarting replay, MIDM, EPSL attacks. Incapable of rendering MA and anonymity features.
LACP-SG	Specialized hardware is required to accomplish the PUF-based AC process. In the future, we will use the AEAD schemes for designing the blockchain-enabled authentication frameworks.

Authenticated encryption with associative data (AEAD), lightweight cryptography (LWC), advance encryption standard (AES), mutual authentication (MA), perfect forward secrecy (PFS), exclusive-OR (XOR), bi-linear paring (BP), elliptic curve cryptography (ECC), authentication and key exchange (AKE), physical unclonable function (PUF), secure hash algorithm (SHA).

**Table 2:** Notations used in LACP-SG.

Notation	Description
$SM_{i}$, $SP_{k}$	Smart meter (SM) and Service Provider (SP), respectively
PUF, CH, *R*	Physically unclonable function, challenge, and response, respectively
$CP_{SP_{k}}$	Common parameter of SP, which is known only to SP
$TID_{SM_{i}}$	Temporary-Identity of smart meter (SM)
$ID_{SM_{i}}$, $ID_{SP_{k}}$, $K_{SP_{k}}$	Real-Identity SM, SP, and secret key of SP
CT and $AP_{tag}$	Ciphertext and authentication parameter (Tag)
PT and $AP_{tag}^{\prime }$	Plaintext and authentication parameter (Tag)
$TS_{1}$, $TS_{2}$	Timestamps in LACP-SG’s AC phase
$T_{mrc}$, $T_{dly}$	Received and maximum delay time of a message
$AD_{1}$, $AD_{2}$	designates the associative data
$N_{1}$, $N_{2}$, $N_{3}$	Signifies the nonce or initialization vector
$E_{K}\left(msge\right)$, $D_{K}\left(msge\right)$	designates COMET based encryption/decryption of message “msge” employing secret key
Gen(·), HD, Rep(·)	Signifies FE based key production, helper data, and key re-production function, respectively
$RN_{1}$, $RN_{2}$, $RN_{3}$	designates the random numbers
A, ‖, H(.), ⊕,	Signifies attacker/adversary, concatenation, hash-function, and XOR, respectively
Adv, INT−CTXT	“Advantage of A and ciphertext integrity”
OPRP−CPA	“Online pseudo-random permutation chosen-plaintext attack”

**Table 3:** ROM-based queries.

Query	Purpose
$Execute(\Psi_{SM_{i}}^{t2},\Psi_{SP_{k}}^{t3})$	Perpetration of this query enables A to seize all the transmitted messages between $SM_{i}$ and $SP_{k}$.
$Send(\Psi^{t},Msg)$	Perpetration of this query enables A to yield an active attack by dispatching a message Msg to $\Psi^{t2}$ and $\Psi^{t1}$ also respond to Msg accordingly.
$Reveal(\Psi^{t})$	Perpetration of this query enables A to get the shared SEK, utilized to guarantee the secure transmission between $\Psi^{t1}$ and its interrelated entity.
$CorruptSM(\Psi_{SM_{i}}^{t2})$	Perpetration of this query helps A to acquire the secret/private parameters loaded in the storage of $SM_{i}$ by operating PA attack.
$Test(\Psi^{t})$	Perpetration of this query enables A to ascertain whether the guessed SEK is licit or random output, just like the outcome of a flipped coin, say *C*.

**Table 4:** Time complexity of different cryptographic operations.

Notations	Operation	Time on R-Pi3	Time on ${\mathrm{SP}}_{k}$
$T_{ecc}$	ECC-based point multiplication	2.70 ms	0.705 ms
$T_{en}$	Symmetric key encryption	0.41 ms	0.015 ms
$T_{eca}$	ECC-based point addition	0.134 ms	0.007 ms
$T_{H}$	One-way hash function (16 bytes)	0.345 ms	0.039 ms
$T_{HE}$	Esch256 one-way hash function (32 bytes)	0.330 ms	0.032 ms
$T_{pu}$	Physical-unclonable-function	0.49 μs	-
$T_{CO}$	COMET	0.349 ms	0.041 ms
$T_{rep}\approx T_{ecc}$	Bio-metric key generation and reproduction	2.70 ms	0.705 ms

Time complexities are computed on Quad-core Raspberry Pi-3 (R-Pi3) with CPU @1.2 GHz, and 1GB of RAM″ and “Core(TM) i7-6700 system with CPU @3.40 GHz, and RAM 8 GB” to simulate $SM_{i}$ $SP_{k}$, respectively.

**Table 5:** Security comparison.

Features	Chaudhry et al. [30]	Bera et al. [29]	Bera et al. [34]	Mehmood et al. [20]	Kumar et al. [11]	Chaudhry et al. [35]	LACP-SG
PrI	✓	✓	✓	×	✓	✓	✓
DIMP	×	✓	✓	✓	×	×	✓
SPI	✓	✓	✓	✓	✓	×	✓
DCA	×	✓	✓	✓	✓	×	✓
MIDM	✓	✓	✓	×	×	✓	✓
DeS	✓	×	×	✓	✓	✓	✓
DoS	✓	✓	✓	×	✓	✓	✓
RA	✓	✓	✓	×	✓	✓	✓
SEKS	✓	✓	✓	×	×	×	✓
EPSL	✓	✓	×	×	×	✓	✓
ROM	✓	✓	✓	×	✓	✓	✓
MA	✓	✓	✓	✓	×	✓	✓
SCER	×	✓	✓	✓	✓	×	-

SCER: secure certificate computation; DCA: device capture attack; ✓: indicates the supported functionality; ×: represents the functionality is not available.

**Table 6:** Communication overhead comparison.

AC Protocol	Disseminated Messages During AC Phase	Total (Bits)
Bera et al. [29]	$SM_{i}\overset{1120}{\to }SP_{k}/GS\overset{1376}{\to }D_{k}/SM_{i}\overset{288}{\to }SP_{k}$	2784
Chaudhry et al. [30]	$SM_{i}\overset{832}{\to }SP_{k}\overset{512}{\to }SM_{i}$	1344
Bera et al. [34]	$D_{k}/SM_{i}\overset{864}{\to }SP_{k}/GS\overset{1216}{\to }D_{k}/SM_{i}\overset{288}{\to }SP_{k}$	2368
Kumar et al. [11]	$SM_{i}\overset{512}{\to }SP_{k}\overset{672}{\to }SM_{i}\overset{192}{\to }SP_{k}$	1376
Chaudhry et al. [35]	$SM_{i}\overset{832}{\to }SP_{k}\overset{832}{\to }SM_{i}$	1664
Mehmood et al. [20]	$SM_{i}\overset{672}{\to }SP_{k}\overset{672}{\to }SM_{i}$	1344
LACP-SG	$SM_{i}\overset{544}{\to }SP_{k}\overset{662}{\to }SM_{i}$	1206

**Table 7:** Computational overhead comparison.

Protocol/Scheme	${\mathrm{SM}}_{i}$ Side	${\mathrm{SP}}_{k}$ Side	Total Time
Bera et al. [29]	$11T_{H}+4T_{ecc}+T_{eca}$	$11T_{H}+4T_{ecc}+T_{eca}$	$22T_{H}+8T_{ecc}+2T_{eca}\approx 17.82$ ms
Chaudhry et al. [30]	$4T_{H}+5T_{ecc}+T_{eca}$	$4T_{H}+4T_{ecc}+T_{eca}$	$8T_{H}+9T_{ecc}+2T_{eca}\approx 17.93$ ms
Bera et al. [34]	$9T_{H}+2T_{en}+2T_{ecc}+T_{eca}$	$9T_{H}+2T_{en}+2T_{ecc}+T_{eca}$	$18T_{H}+4T_{en}+4T_{ecc}+2T_{eca}\approx 11.12$ ms
Kumar et al. [11]	$6T_{H}+2T_{ecc}$	$6T_{H}+2T_{ecc}$	$12T_{H}+4T_{ecc}\approx 14.42$ ms
Chaudhry et al. [35]	$4T_{H}+5T_{ecc}+2T_{eca}$	$4T_{H}+5T_{ecc}+3T_{eca}$	$8T_{H}+9T_{ecc}+5T_{eca}\approx 18.79$ ms
Mehmood et al. [20]	$4T_{H}+5T_{ecc}+2T_{eca}$	$4T_{H}+5T_{ecc}+2T_{eca}$	$8T_{H}+10T_{ecc}+4T_{eca}\approx 18.18$ ms
LACP-SG	$2T_{HE}+2T_{co}+T_{rep}+T_{pu}$	$5T_{HE}+2T_{co}$	$7T_{HE}+4T_{co}+T_{rep}+T_{pu}\approx 4.34$ ms

## Data Availability

Not applicable.
